# 2022 Acknowledgment of *mBio* Invited Editors

**DOI:** 10.1128/mbio.03100-22

**Published:** 2022-12-20

**Authors:** Arturo Casadevall

**Affiliations:** a Johns Hopkins Bloomberg School of Public Health, Baltimore, Maryland, USA

## EDITORIAL

On behalf of the editors of *mBio*, I gratefully acknowledge the following individuals, who served as invited editors for the journal in 2022. While not members of the Board of Editors, invited editors play an important role in the review process. Invited editors are experts in their fields of research who contribute an additional level of quality to the review process. An editor may assign a paper to an invited editor when they would like to have an additional expert opinion of the reviews or when the subject area falls outside the editor’s primary area of expertise.

Mohamed Abdel-Mohsen
Niyaz Ahmed
David R. Andes
Jason R. Andrews
Jonathon P. Audia
Frank O. Aylward
Fernando Baquero
Christopher Barnes
Himanshu Batra
Patrik M. Bavoil
Anke Becker
Megan Bergkessel
Marc J. Bonten
David G. Bourne
Axel A. Brakhage
Benjamin Brennan
Margo A. Brinton
Eric D. Brown
Stanley Brul
Edward M. Campbell
Samuel K. Campos
Jason A. Carlyon
Jamie Cate
Yunrong Chai
Georgios Chamilos
Patricia A. Champion
Arnab Chatterjee
Mallory J. Choudoir
Jan Claesen
Phillip Cleves
Beatrice Clouet-d'Orval
Pierre Cornelis
Andrea L. Cox
Robert Craigie
Bryan R. Cullen
Anthony L. Cunningham
Christina A. Cuomo
Cameron R. Currie
Christiane Dahl
Sophie E. Darch
Sanjay A. Desai
Santosh Dhakal
Dirk P. Dittmer
Jaquelin P. Dudley
Mary Dunlop
Nels C. Elde
Melissa Ellermann
Mohamed Y. El-Naggar
Michael Emerman
Joanne Engel
Mary K. Estes
Paul D. Fey
Suzanne M. J. Fleiszig
Karl Forchhammer
Adriana Forero
Simon J. Foster
Karen M. Frank
Matthew B. Frieman
Xinmiao Fu
Isabelle Fudal
Takema Fukatsu
Adolfo García-Sastre
Pallavi Ghosh
Daniel E. Goldberg
Susan S. Golden
Mark Goulian
Scott S. Grieshaber
Haitao Guo
Benjamin G. Hale
Brian K. Hammer
Cara H. Haney
Reuben S. Harris
Alex Hayward
Patrick Hearing
Jeffrey P. Henderson
David R. Hendrixson
Kent L. Hill
Ann M. Hirsch
Alexander Horswill
Peter M. Howley
James Imlay
Christine Jacobs-Wagner
Swati Jain
Anthony A. James
Urs Jenal
Dong-Yan Jin
Hailing Jin
Jeremy Phillip Kamil
Melissa M. Kendall
Frank Kirchhoff
Matthew Kirk
Tino Krell
Richard J. Kuhn
Stephanie N. Langel
Shan-Lu Liu
William Ludington
Elizabeth K. Mallott
Anthony Maresso
Christopher W. Marshall
Jere W. McBride
Shonna M. McBride
Rachel McLoughlin
Andrew Moeller
Karl Münger
Lishomwa C. Ndhlovu
Karen M. Ottemann
Tracy Palmer
Kai Papenfort
Colin R. Parrish
Andrew Pekosz
Owen Pornillos
Christopher Power
Janet Quinn
Tracy Lyn Raivio
David Rasko
Felix A. Rey
Jeremy M. Rock
Christopher Daniel Andrade Rodrigues
Andrew J. Roe
Morgane Rolland
Mikiyasu Sakanaka
Christian Santos-Medellín
Jeffrey W. Schertzer
Margaret A. Scull
Mohamed G. Seadawy
Amir Sharon
Liang Shi
Sujan Shresta
Robert F. Siliciano
Thomas J. Silhavy
Lyle A. Simmons
Donald Sodora
Filipa L. Sousa
Mark D. Stenglein
Marulasiddappa Suresh
Michael G. Surette
Curtis A. Suttle
Marc Swidergall
Michiko E. Taga
Nick Talbot
Benjamin R. tenOever
David L. Thomas
Kürsad Turgay
B. Gillian Turgeon
Giel G. van Dooren
Ivona Vasile-Pandrea
Joe Vogel
Matthew K. Waldor
Mark R. Walter
Matthew Wargo
Paula I. Watnick
Robert O. Watson
Richard J. Webby
James Weger-Lucarelli
Daniela Weiskopf
Brian Weiss
David S. Weiss
Matthew D. Weitzman
Haitao Wen
Nathan John Weyand
Rachel Whitaker
Craig B. Wilen
William Wiley Navarre
Angela Wilks
Daniel Wilson
David Wilson
Richard A. Wilson
Christiane Wolz
Li Wu
Ellen Yeh
Zhi-Ming Zheng

